# Fluoxetine minimally affects hearing loss but induces gene expression changes in the cochlear nuclei after noise exposure

**DOI:** 10.1371/journal.pone.0341746

**Published:** 2026-02-03

**Authors:** Hyun-Ju An, Sujin Choi, Soonchul Lee, Hyunjeong Yeo, So Young Kim

**Affiliations:** 1 Department of Orthopaedic Surgery, CHA Bundang Medical Center, CHA University School of Medicine, 335 Pangyo-ro, Bundang-gu, Seongnam-si, Gyeonggi-do, Republic of Korea; 2 Department of Anatomy and Cell Biology, Seoul National University College of Medicine, Seoul, Republic of Korea; 3 Sensory Organ Research Institute, Seoul National University Medical Research Center, Seoul, Republic of Korea; Morsani College of Medicine, University of South Florida, UNITED STATES OF AMERICA

## Abstract

**Purpose:**

This study investigated the effects of fluoxetine on noise-induced injuries to the cochlea and auditory nerve, with a focus on its impact on perineuronal nets (PNNs) and gene expression changes in the ventral cochlear nuclei (VCN).

**Methods:**

Sprague-Dawley rats were exposed to white noise at 115 dB SPL for 3 hours per day over 3 weeks. After measuring auditory brainstem response (ABR) thresholds, rats were treated with fluoxetine (10 mg/kg) for 19 days. Four groups were included (vehicle, fluoxetine, noise + vehicle, and noise + fluoxetine; n = 10 per group). ABR measurements, analysis of extracellular baskets in cochlear ribbon synapses and PNNs, and RNA sequencing of the VCN were performed.

**Results:**

Following fluoxetine treatment, noise-exposed rats (noise + fluoxetine group) showed hearing thresholds comparable to those in the noise + vehicle group. Both noise-exposed groups exhibited cochlear hair cell loss and disorganization. Extracellular baskets surrounding cochlear ribbon synapses were significantly reduced in noise + vehicle rats and were not restored in noise + fluoxetine rats. Aggrecan expression in the VCN was reduced in the noise + fluoxetine group. RNA sequencing revealed upregulation of genes including *Mal, Fos, Rapgef3, Papss2, Adamts4,* and *Heph,* and downregulation of genes such as *Pde5a, Kcnma1, Nr4a1, Dlgap3, Slc18a2,* and *Dgkg*.

**Conclusion:**

Fluoxetine exerted only modest, 4 kHz‑restricted improvements in ABR thresholds and did not restore cochlear structure or normal hearing following noise‑induced hearing loss. However, it induced distinct transcriptional alterations in the VCN and modulated the extracellular environment, suggesting a potential role in neural remodeling rather than direct auditory restoration.

## 1. Introduction

Noise exposure can lead to cochlear neural degeneration and loss of hair cells [1], particularly when it occurs during critical periods of auditory development. While multiple molecular mechanisms contribute to noise-induced hearing loss, effective clinical treatments remain unavailable [2–6]. The development of primary auditory afferents continues through the postnatal period, which is completed approximately 2–3 weeks after birth [7]. Thus, noise exposure during the critical period of hearing onset can induce more severe degenerative changes in the cochlea and auditory nervous system.

Fluoxetine, a widely used selective serotonin reuptake inhibitor (SSRI), has demonstrated effects beyond its antidepressant properties. Chronic fluoxetine treatment re‑opens critical‑period‑like plasticity, normalizes inhibitory circuitry, and promotes structural and functional remodeling in sensory cortices after injury [8]. Importantly, systemic SSRIs can cross the blood–labyrinth barrier and serotonergic receptors and transporters are expressed in the cochlea and auditory brainstem, suggesting that serotonin modulators can directly or indirectly influence auditory processing [9]. In animal models, serotonergic drugs have been reported to alter cochlear blood flow, modulate outer hair cell motility, and affect central auditory neuron excitability, supporting a potential role for serotonin signaling in noise‑related auditory pathophysiology [10]. Recent studies suggest that fluoxetine enhances neural plasticity in the visual and auditory cortices through pathways involving the activation of tyrosine kinase receptor B (TrkB) and its ligand, brain-derived neurotrophic factor (BDNF) [11–13]. In the hippocampus, fluoxetine-induced neurogenesis is thought to alleviate depressive behaviors via serotonin receptor pathways [14]. TrkB is a receptor for BDNF [15]. In addition to the serotonin pathway, fluoxetine acts by binding with the transmembrane domain of TrkB [15,16]. In addition to its neurotrophic effects in the hippocampus, the activation of TrkB reportedly improves cochlear injury induced by noise exposure [17]. Although the effects of TrkB in noise-induced hearing loss have been suggested to be mediated by activation of the TrkB pathway, there is a lack of knowledge on its effects on the auditory nerve and downstream molecular players related to the effects of TrkB in the auditory system.

Neural plasticity driven by fluoxetine is associated with changes in PNNs, a specialized extracellular matrix primarily found in parvalbumin-expressing interneurons [18–21]. The attenuation of PNNs induces critical period-like neural plasticity [19]. The attenuation of PNNs has been shown to restore critical period-like plasticity in the visual system [22]. The auditory nervous system changes the PNNs according to peripheral sensory deficits [23,24]. For example, the attenuation of PNNs was presented in the primary auditory cortex after noise exposure [25]. On the other hand, PNNs expression increases with age in the inferior colliculi [26–28], which indicates the consolidated auditory neural circuit and resistance of auditory neural plasticity in aged subjects. Thus, alleviation of PNNs may restore neural plasticity in the central auditory system.

Our previous study reported that fluoxetine treatment in noise-exposed rats partially restored auditory processing and extracellular matrix-related gene expression, including aggrecan (ACAN) and BDNF, in the auditory cortex and hippocampus [29]. However, its effects on lower auditory centers of cochlear nuclei and cochlea were not explored. The cochlear inner hair cells, which contains an extracellular basket analogous to PNNs [30], was predicted to be modulated by fluoxetine. Therefore, we hypothesized that fluoxetine would modulate TrkB‑dependent plasticity in the peripheral and central auditory pathways after early‑life noise exposure. Specifically, we tested whether fluoxetine improves auditory brainstem response (ABR) thresholds and preserves cochlear hair cells and extracellular baskets, and induces transcriptional changes consistent with enhanced central plasticity.

## 2. Materials and methods

### 2.1 Animal models

All animal protocols were reviewed and approved by the CHA University Medical School Institutional Animal Care and Use Committee (IACUC #170162). All methods were carried out in accordance with the guidelines and regulations of the Institutional Animal Care and Use Committee of CHA University Medical School**.** The rats were raised at 20–24 °C, 70–75% humidity, a 12-h light/dark cycle, and a cage with two rats. We purchased Specific Pathogen-Free (SPF) Sprague–Dawley (SD) rats from Koatech and housed them in an SPF animal facility for the duration of the study.

**Fig 1:**
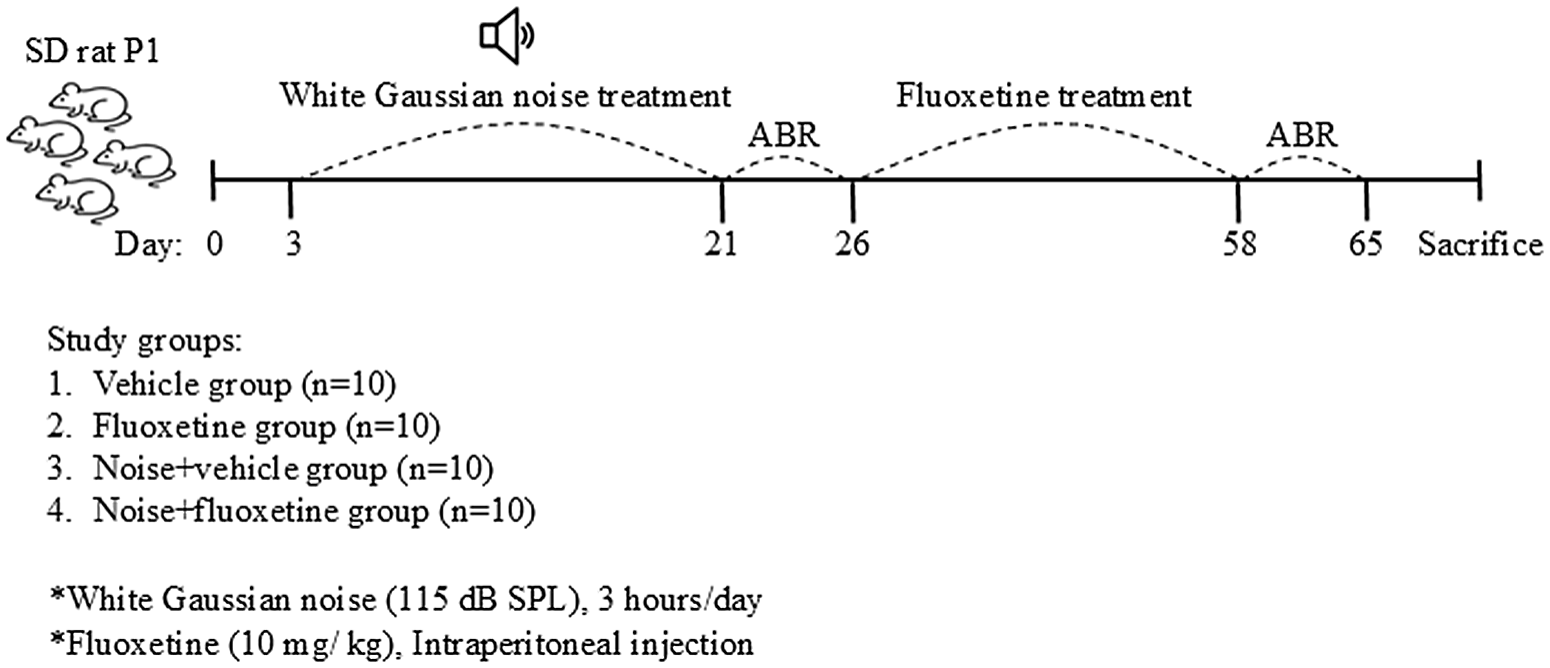
Schematic representation of the animal experiment schedule. Postnatal day 1 (P1) rats were divided into four groups. Rats in the noise and noise + fluoxetine groups were exposed to white noise for 3 weeks. After completing noise exposure, auditory brainstem response (ABR) measurements were performed to assess auditory function. Following ABR assessment, rats in the noise + fluoxetine group received intraperitoneal injections of fluoxetine at a dose of 10 mg/kg for three weeks. After the treatment period, ABR was measured again to evaluate auditory recovery. Finally, the rats were sacrificed, and the cochleae and cochlear nuclei were dissected for further analysis.

Postnatal day 3–5 Sprague–Dawley rats were classified into four groups (n = 10 for each group, Fig 1): (1) the no noise exposure and normal saline injection group (vehicle rats), (2) the no noise exposure and fluoxetine injection group (fluoxetine rats), (3) the noise exposure and normal saline injection group (noise+vehicle rats), and (4) the noise exposure and fluoxetine injection group (noise+fluoxetine rats). Auditory brainstem response threshold (ABRT) tests were conducted after noise exposure (pretreatment) and after fluoxetine administration (posttreatment) in all the rats (n = 10 for each group). Subjects were randomly assigned to experimental groups at postnatal day 1, and all outcome assessments, including ABR measurements and histological analyses, were performed by investigators blinded to group allocation.

After the ABR tests were completed, the rats were sacrificed, and the VCN of two rats per group were collected and analyzed via RNA sequencing. We utilized CO₂ gas for euthanasia, adhering to the IACUC guideline. The method involved a gradual fill of CO₂ gas into the chamber to minimize distress, with a flow rate of 30% of the chamber volume per minute. The animals were kept in the CO₂ chamber for 15–20 minutes to ensure complete euthanasia. No additional anesthetics or injections were used prior to euthanasia.

### 2.2 Noise exposure

White noise (2–20 kHz, 115 dB SPL) was applied to the noise and noise+fluoxetine rats for 3 hours per day for 5 days per week for 3 weeks. Noise was delivered through a soundproof chamber using a free-field electrostatic speaker, which was located on top of the chamber [23]. The rats were awake during noise exposure. The vehicle and fluoxetine rats were subjected to background noise of 40–50 dB SPL. The rats were weaned at postnatal day 21.

### 2.3 Fluoxetine administration

After completion of the noise exposure and behavioral tests, the fluoxetine and noise+fluoxetine rats were intraperitoneally administered 10 mg/kg fluoxetine for 19 days. The dose of fluoxetine used was adapted from a previous study [31]. Vehicle and noise+vehicle rats were intraperitoneally administered an equivalent volume of normal saline during identical periods (19 days).

### 2.4 Auditory brainstem response

At the end of the noise exposure, the ABRTs at 4, 8, 16, and 32 kHz were evaluated via the SmartEP system (Intelligent Hearing System, Miami, FL, USA), as described in previous studies [32]. The rats were anesthetized after intraperitoneal injection of Zoletil (40 mg/kg) and xylazine (10 mg/kg). Tone bursts (duration: 1562 µs; envelope: Blackman; stimulation rate: 21.1/s; 1024 sweeps) at 4, 8, 16, and 32 kHz were delivered via plastic earphones, which were inserted into the external auditory canal. The intensity of the tones was lowered from 90 dB SPL with 10 dB SPL intervals to 10 dB SPL intervals. The lowest tone intensity with evoked wave III (approximately 2–4 ms) was identified as an auditory threshold. Out of 10 rats, bilateral hearing measurements were possible in 2 rats, resulting in a total of 12 ears with ABR recordings.

### 2.5 Immunohistochemistry

A paraffin block was made with whole-brain and cochlear samples. The cochleae were decalcified in EDTA solution before being embedded. The paraffin block was sectioned into 6 µm thick sections via a rotary microtome. The sectioned slices were mounted on coated glass slides. Immunohistochemistry was conducted via SuperBioChips via a Link48 automated immunostainer and an Envision DAB (Agilent, CA, United States) as previously described [33]. All antibodies used in this study were using Tissue Microarray (TMA) slides [33].

The slides were incubated with primary antibodies (mouse monoclonal [C-5] IgG2a kappa anti-myosin7a [sc-74516, Santa Cruz], 1:30; mouse monoclonal [E-4] IgG1 kappa anti-sox2, [sc-365823, Santa Cruz], 1:100; rabbit monoclonal [EPR1292] IgG anti-BDNF [ab108319, Abcam], 1:5000; polyclonal goat IgG anti-TrkB [AF1494, R&D systems], 1:150; rabbit monoclonal [EPR25743–59] IgG anti-brevican (BCAN) [ab285162, Abcam], 1:1000; polyclonal IgG anti-TRKB [AF1494, R&D systems], 1:500; and rabbit multiclonal [RM2036] IgG anti- ACAN [ab315486], 1:100) overnight at 4°C. After the samples were washed in PBS three times, a biotinylated secondary antibody was applied, and the samples were incubated for 30 minutes at room temperature. The ABC Elite ABC reagent was subsequently added, and the mixture was incubated for 30 minutes at room temperature. After the samples were washed in PBS, DAB substrate was applied for 2–7 minutes. After the samples were washed in PBS, counterstaining was conducted with hematoxylin. Dehydration was performed with a series of ethanol solutions. The slide was mounted with a cover slip. The slides were examined under a bright field microscope. The counting of positive cells was conducted via the QuPath program. After color deconvolution of hematoxylin and DAB channels, regions of interest (ROI) were manually drawn to include the spiral ganglion and VCN and exclude background or damaged tissue. Positive cell detection was performed with the positive cell detection command. Positive cells were quantified using QuPath and expressed as the number of positive cells per mm², followed by statistical analysis using ANOVA. The quantification of the signal intensity of TrkB, aggrecan, and brevican in cochlea was performed via the ImageJ program. For each section, images were acquired under identical exposure and gain settings, converted to 8‑bit grayscale, and background‑subtracted. Mean gray value was measured within manually defined ROI, corresponding to the extracellular baskets of cochlear ribbon synapses, and values were normalized to the area (µm²) or to the mean intensity of the control group, followed by statistical analysis with ANOVA. All images were processed and quantified under blinded conditions using the same coding procedure as for cell counts.

Cochlear whole mount immunostaining was conducted for myosin7a and sox2. Decalcified cochleae were carefully dissected. Dissected cochleae were washed with PBS and then blocked in 4% donkey serum with 0.2% Triton for 1 hour. Primary antibodies (mouse monoclonal [C-5] IgG2a kappa anti-myosin7a [sc-74516, Santa Cruz], 1:250; mouse monoclonal [E-4] IgG1 kappa anti-sox2, [sc-365823, Santa Cruz], 1:250) were incubated overnight. After 3 washes in 0.2% Triton PBS, the sections were incubated with secondary antibodies (Alexa Fluor 594-conjugated donkey anti-mouse IgG [A21203, Thermo Fisher Scientific], 1:500) for 2 hours. After 3 washes, the slides were mounted with mounting solution. A fluorescence microscope was used to examine the cochlear whole mount immunostained slides.

### 2.6 RNA sequencing and analysis

RNA isolation and RNA sequencing were conducted via Ebiogen® according to the manufacturer’s protocol [29]. Total RNA was extracted using TRIzol (Invitrogen), and RNA quality and concentration were evaluated with TapeStation 4000 (Agilent Technologies) and ND-2000 spectrophotometer (Thermo). Library construction was performed with the QuantSeq 3′ mRNA-Seq Library Prep Kit (Lexogen) following standard protocols. Sequencing was conducted on the Illumina NextSeq 550 platform with single-end 75 bp reads.

Raw reads were aligned to the reference genome using Bowtie2. Gene-level read counts were obtained with Bedtools, and normalization and differential expression analysis were carried out using the TMM-CPM method implemented in EdgeR (Bioconductor, R v4.0). Differentially expressed genes (DEGs) were defined as those with a fold change > 2.0, P < 0.05, and normalized expression value > 4. Functional annotation of DEGs was performed using DAVID and literature databases. Visualization and data mining were conducted with ExDEGA software (Ebiogen).

### 2.7 Statistical analysis

Group differences in ABR thresholds, gene expression, and immunostaining measures were assessed by ANOVA followed by multiple comparison using Tukey tests. Paired and independent t tests were used to compare ABR thresholds within and between groups, respectively. Significance was set at P < 0.05. All analyses were performed using GraphPad Prism 8.0 and SPSS 21.0.

## 3. Results

### 3.1 Increased hearing threshold in noise+vehicle and noise+fluoxetine rats

**Fig 2:**
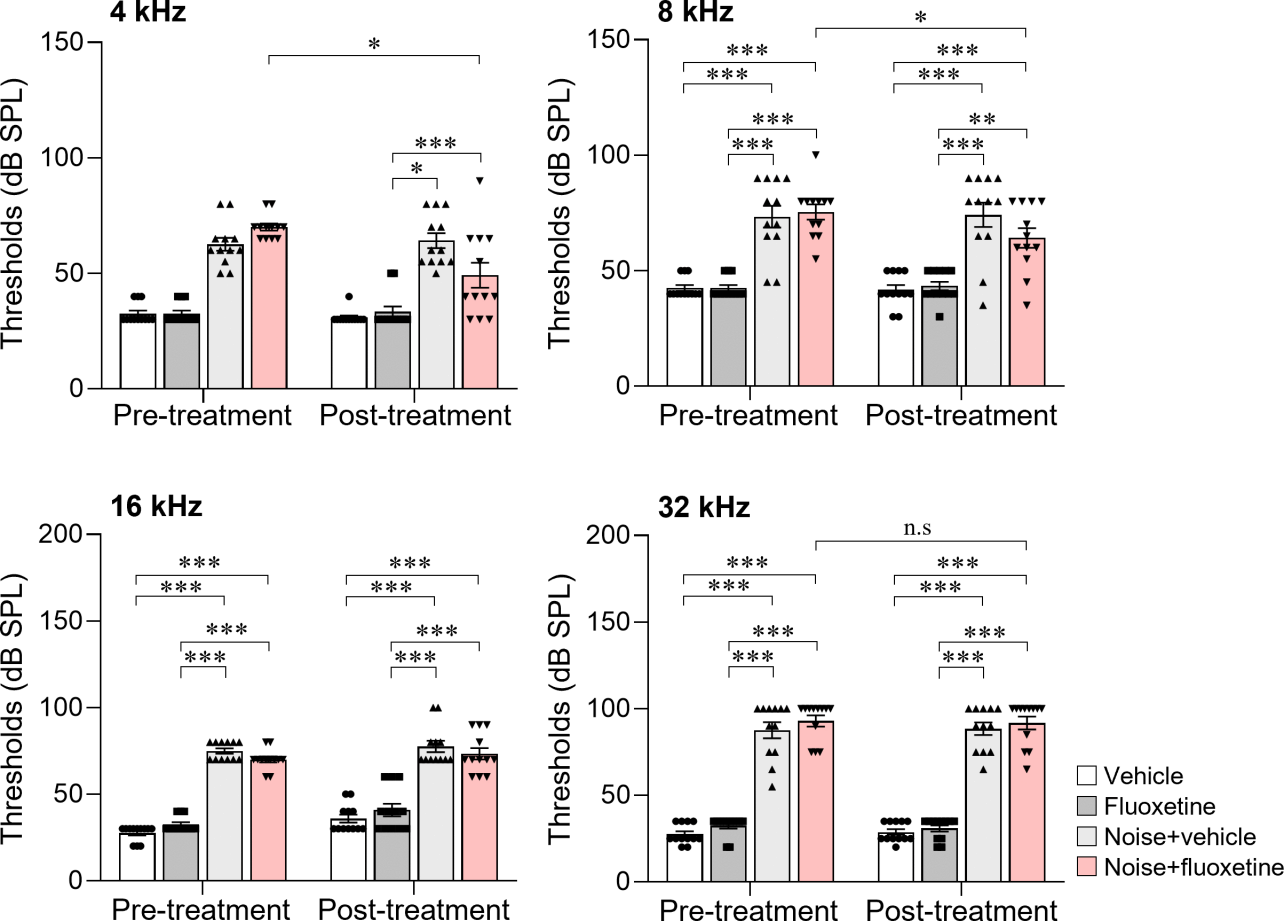
Changes in auditory brainstem response (ABR) thresholds at 4, 8, 16, and 32 kHz before (pretreatment) and after (posttreatment) fluoxetine or saline treatment (n = 10 per group). Posttreatment ABR thresholds in the fluoxetine-treated group (noise + fluoxetine) were significantly lower at 4 and 8 kHz compared to the pretreatment values. Significant differences were observed between groups (*P < 0.05, independent t test) and within groups (pretreatment vs posttreatment, **P < 0.05, paired t test).

All rats exposed to noise exhibited elevated auditory thresholds at 4, 8, 16, and 32 kHz (pretreatment, Fig 2). After 19 days of treatment with either fluoxetine or an equivalent volume of saline, ABR tests were repeated (posttreatment). Compared to pretreatment values, the noise+fluoxetine group showed slightly reduced auditory thresholds, which were limited to 4 and 8 kHz. In the noise+fluoxetine group, the average ABR threshold at 4 kHz was 70.00 ± 5.22 dB SPL before treatment and 49.16 ± 18.81 dB SPL after treatment (95% confidence interval [CI] = 8.28–33.38, R^2^ = 0.548, P = 0.004, paired t-test). At 8 kHz, the threshold decreased from 75.42 ± 11.37 dB SPL to 64.17 ± 14.75 dB SPL (95%CI = 1.28–21.22, R^2^ = 0.400, P = 0.03, paired t-test). No significant changes in ABR thresholds at 16 or 32 kHz were observed in any group. Compared with the noise-only group, the noise+fluoxetine group exhibited lower ABR thresholds at 4 kHz (95%CI = 1.880–28.12, R^2^ = 0.204, P = 0.03, unpaired t-test), but not at 8 kHz (95%CI = −6.789–10.00, R^2^ = 0.090, P = 0.15, unpaired t-test).

### 3.2 Hair cells and extracellular baskets of cochlea after fluoxetine administration

**Fig 3:**
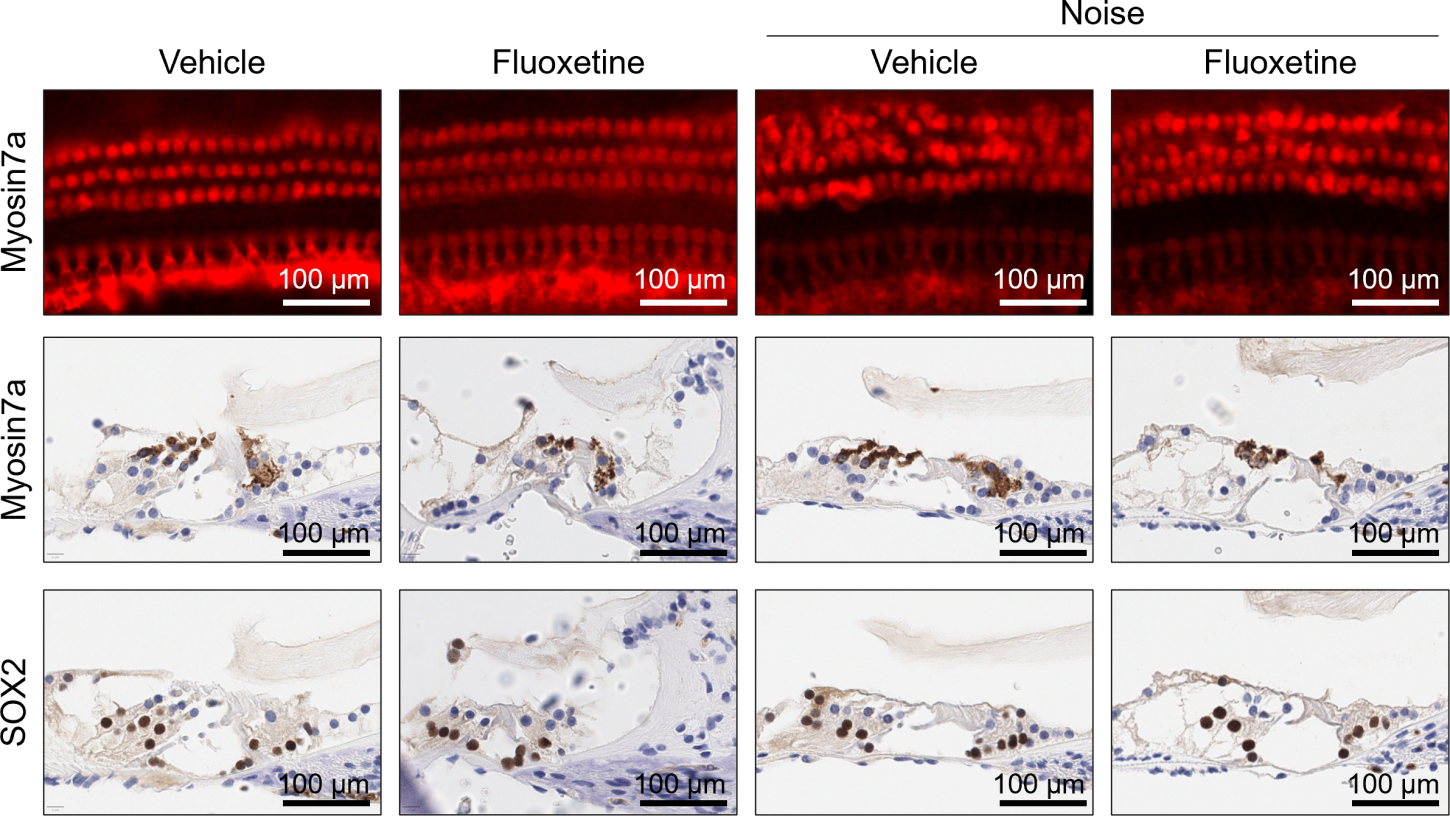
Noise exposure induced injuries to cochlear outer hair cells, which were not mitigated by fluoxetine treatment. **(A)** Whole-mount cochlear immunofluorescence staining for myosin7a showed loss and disorientation of outer hair cells in noise-exposed rats. Both noise and noise+fluoxetine groups showed comparable outer hair cell loss. **(B)** Cochlear immunohistochemistry for myosin7a (outer and inner hair cells) and sox2 (supporting cells) demonstrated structural differences among the four groups. Outer and inner hair cells appeared smaller or shorter in noise-exposed rats (noise and noise + fluoxetine groups) compared to control and fluoxetine-only groups. The number of supporting cells remained consistent across all groups.

**Fig 4:**
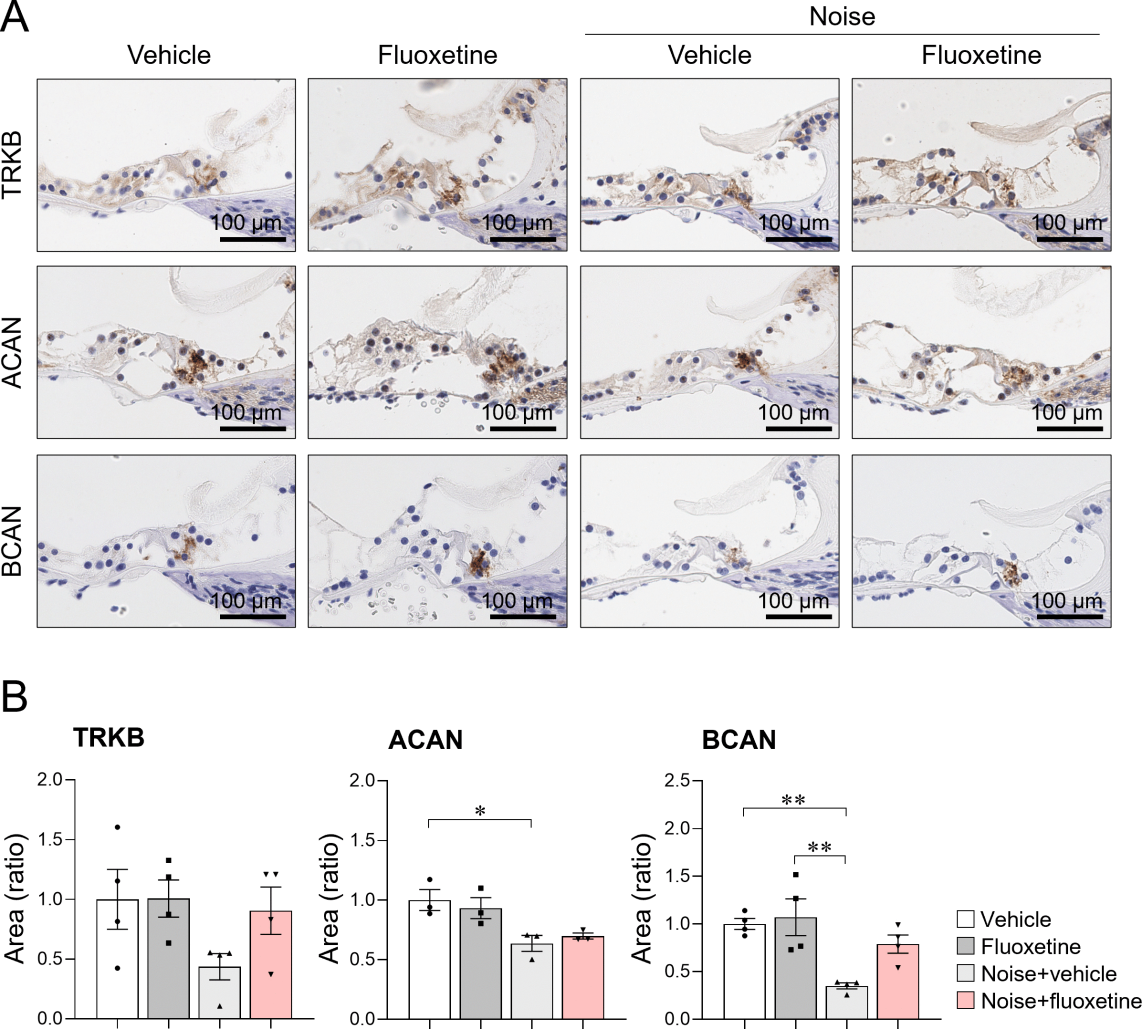
Noise exposure decreased the expression of TrkB, aggrecan (ACAN), and brevican (BCAN) in the cochlea. Fluoxetine treatment following noise exposure (noise + fluoxetine group) partially increased their expression levels, but levels did not return to those of vehicles. The graphs depict the relative area of cochlear ribbon synapses, reflecting the expression of each protein. Significant differences were observed between vehicle and noise-exposed rats (*P < 0.05) and between noise-exposed and noise + fluoxetine-exposed rats (**P < 0.05) according to the one‑way ANOVA followed by Tukey test.

In the noise+vehicle group, cochlear hair cells in the middle turn were disoriented and lost (Fig 3). Similarly, the noise+fluoxetine group exhibited comparable levels of hair cell disorientation and loss. The expression of ACAN and BCAN was observed in the extracellular baskets surrounding inner hair cells, with differential expression among groups (Fig 4; R^2^ = 0.676, P = 0.003 for BCAN and R^2^ = 0.696, P = 0.018 for ACAN, ANOVA). Specifically, BCAN expression was significantly lower in noise+vehicle rats compared to vehicle controls (95%CI = 0.179–1.121, P = 0.007, Multiple comparison with Tukey test). Likewise, ACAN expression was reduced in the noise+vehicle group (95%CI = 0.040–0.688, P = 0.030, Tukey test). In contrast, the noise+fluoxetine group showed no significant differences in ACAN or BCAN expression compared to the other groups. TrkB expression was similar across all groups.

### 3.3 Attenuation of perineuronal nets in cochlear nuclei after fluoxetine administration

**Fig 5:**
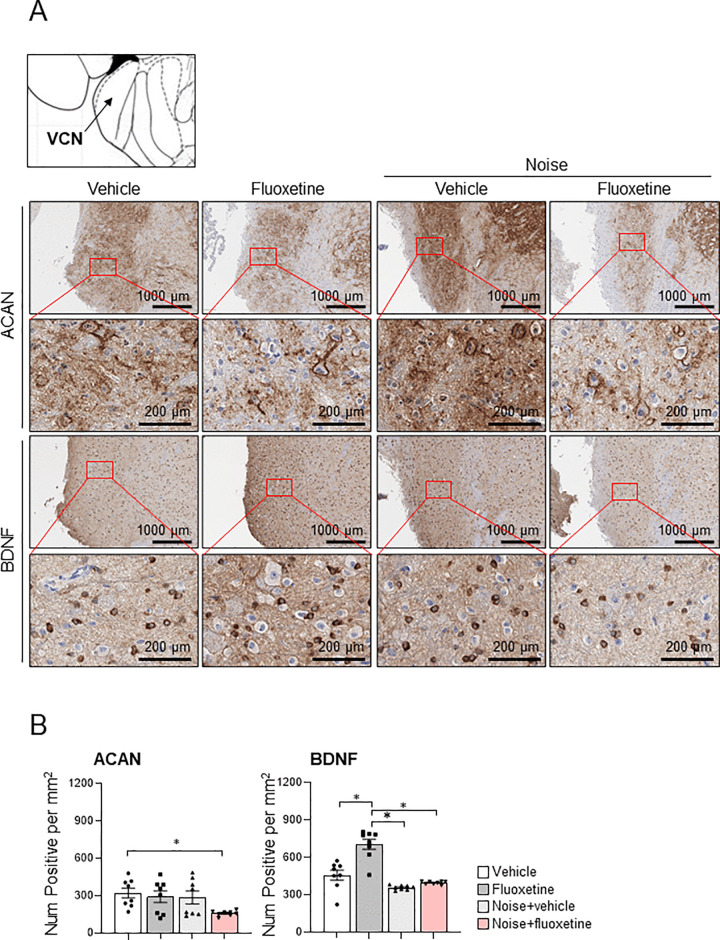
The expression levels of aggrecan (ACAN) and brain-derived neurotrophic factor (BDNF) in the ventral cochlear nucleus (VCN) (A) Representative low‑ and high‑magnification images of ACAN‑ and BDNF‑immunoreactive cells in the VCN from vehicle, fluoxetine, noise-exposed, and noise+fluoxetine groups (bregma −9.68 mm; atlas reference: labs.gaidi.ca/rat-brain-atlas). (B) Quantification of ACAN‑positive PNNs and BDNF‑positive cells in the VCN, expressed as the number of positive cells per mm². Data shown as mean ± SEM. Statistical analysis was performed using one‑way ANOVA followed by Tukey’s post hoc test; *P < 0.05.

To investigate changes in the auditory nerve following noise exposure and fluoxetine treatment, the VCN were analyzed for the expression of ACAN, a principal component of PNNs, and BDNF (Fig 5). In noise+vehicle rats, ACAN expression was comparable to that in vehicle and fluoxetine groups. However, the noise+fluoxetine group exhibited a significantly lower number of ACAN-positive cells in the VCN compared to the other groups (R^2^ = 0.255, P = 0.038, ANOVA; 95%CI = 0.040–0.688, P = 0.039, Tukey test between noise+fluoxetine and vehicle groups). The neurotrophic effect was assessed through BDNF expression. Fluoxetine-only rats showed elevated BDNF expression in the VCN compared to the other groups. However, BDNF expression in noise+fluoxetine rats were not increased compared to noise+vehicle rats.

### 3.4 Differential gene expression and fluoxetine-induced transcriptional responses in the cochlear nuclei

**Fig 6:**
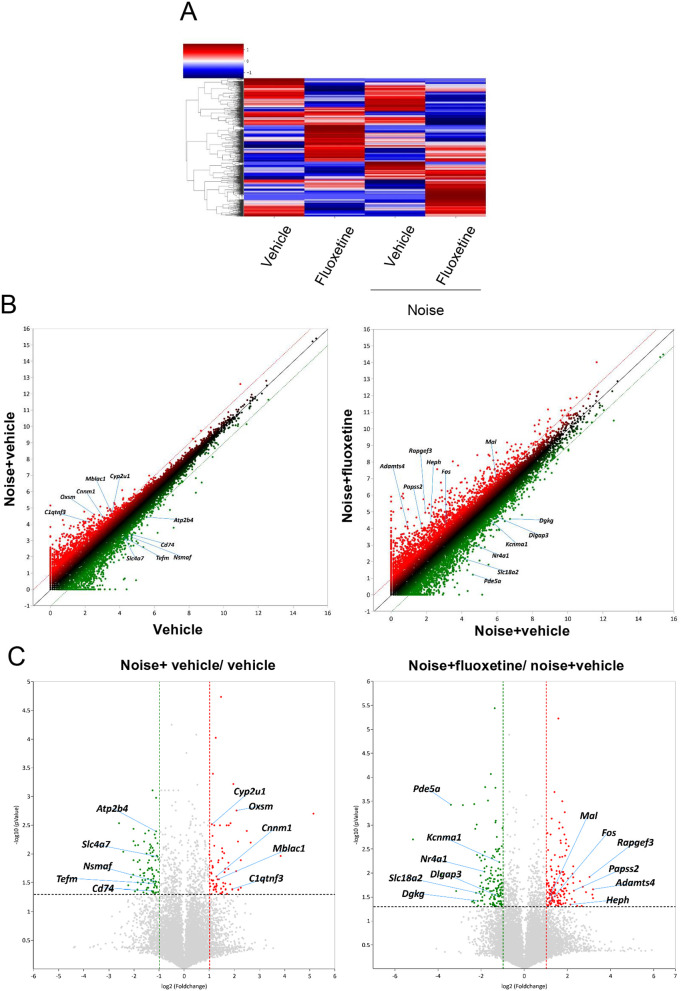
RNA sequencing analysis of the cochlear nuclei revealed differential gene expression and predicted pathways associated with noise exposure and fluoxetine treatment. **(A)** Heatmap analysis shows distinct gene expression profiles among experimental groups. **(B)** Scatter plots illustrate upregulated and downregulated genes in noise+vehicle vs. vehicle, and noise+fluoxetine vs. noise+vehicle comparisons. **(C)** Volcano plots display differentially expressed genes for noise+vehicle vs. vehicle and noise+fluoxetine vs. noise+vehicle.

To investigate the effect of fluoxetine on noise-induced auditory nerve injury through the regulation of specific gene groups, we performed mRNA sequencing of the VCN in rats (Fig 6). Heatmap analysis of DEGs visually demonstrated the expression changes across the groups (Fig 6A). Hierarchical clustering revealed distinct gene expression profiles for each group, with the noise+vehicle and noise+fluoxetine groups showing significant upregulation and downregulation of specific gene clusters compared to the vehicle and fluoxetine-only groups.

Scatter plots and volcano plots of DEG analysis further highlighted these significant expression changes (Fig 6B and 6C). In the noise+vehicle group compared to the vehicle group, genes such as *Cyp2u1* (Cytochrome P450 family 2 subfamily U member 1), *Oxsm* (3-oxoacyl-[acyl-carrier-protein] synthase, mitochondrial), *Cnnm1* (Cyclin M1), *Mblac1* (Metallo-β-lactamase domain-containing protein 1), and *C1qtnf3* (C1q and tumor necrosis factor related protein 3) were notably upregulated. Conversely, *Atp2b4* (ATPase plasma membrane Ca² ⁺ transporting 4), *Slc4a7* (Solute carrier family 4 member 7), *Nsmaf* (Neutral sphingomyelinase activation associated factor), *Tefm* (Transcription elongation factor, mitochondrial), and *Cd74* (Class II major histocompatibility complex-associated invariant chain) were downregulated.

Comparison between the noise+fluoxetine and noise+vehicle groups also revealed significant alterations. Genes such as *Mal* (Myelin and lymphocyte protein), *Fos* (FBJ osteosarcoma oncogene), *Rapgef3* (Rap guanine nucleotide exchange factor 3), *Papss2* (3’-phosphoadenosine 5’-phosphosulfate synthase 2), *Adamts4* (A disintegrin and metalloproteinase with thrombospondin motifs 4), and *Heph* (Hephaestin) were significantly upregulated. In contrast, *Pde5a* (Phosphodiesterase 5A), *Kcnma1* (Potassium calcium-activated channel subfamily M alpha 1), *Nr4a1* (Nuclear receptor subfamily 4 group A member 1), *Slc18a2* (Solute carrier family 18 member A2), *Dlgap3* (Discs large-associated protein 3), and *Dgkg* (Diacylglycerol kinase gamma) were significantly downregulated in the noise+fluoxetine group compared to the noise+vehicle group. In total, 39 genes were identified as upregulated and 52 genes as downregulated DEGs in the noise+fluoxetine group compared to the noise+vehicle group (Table 1 and Table 2).

**Table 1:** List of upregulated genes in the cochlear nuclei of rats treated with noise and fluoxetine compared to noise exposure alone.

Gene symbol	Fold change				p-value					Gene description
	Noise+vehicle/vehicle	Noise+fluoxetine/Noise+vehicle	Fluoxetine/vehicle	Noise+fluoxetine/fluoxetine	Noise+vehicle/vehicle	Noise+fluoxetine/Noise+vehicle	Fluoxetine/vehicle	Noise+fluoxetine/fluoxetine	ANOVA	
*Adamts4*	0.23	9.05	2.39	0.87	0.27	0.02	1.00	1.00	0.02	ADAM metallopeptidase with thrombospondin type 1 motif, 4
*Rapgef3*	0.34	8.11	1.12	2.48	0.08	0.01	1.00	1.00	0.00	Rap guanine nucleotide exchange factor 3, transcript variant X2
*Papss2*	0.78	4.90	1.19	3.19	0.46	0.02	1.00	1.00	0.00	3’-phosphoadenosine 5’-phosphosulfate synthase 2, transcript variant X1
*Heph*	0.43	4.37	2.07	0.91	0.30	0.05	1.00	1.00	0.03	hephaestin, transcript variant X3
*Myo5b*	1.21	3.88	3.25	1.44	0.72	0.02	1.00	1.00	0.02	myosin Vb, transcript variant X3
*Anapc15*	0.34	3.81	1.16	1.10	0.08	0.00	1.00	1.00	0.00	anaphase promoting complex subunit 15, transcript variant X8
*Fam221a*	0.50	3.54	0.34	5.12	0.08	0.03	1.00	1.00	0.00	family with sequence similarity 221, member A, transcript variant X1
*Lima1*	0.47	3.49	1.76	0.94	0.28	0.01	1.00	1.00	0.03	LIM domain and actin binding 1, transcript variant X2
*Plek*	0.60	3.28	0.87	2.29	0.41	0.01	1.00	1.00	0.07	pleckstrin
*Rcor2*	0.72	3.11	1.46	1.54	0.12	0.01	1.00	1.00	0.00	REST corepressor 2, transcript variant X1
*Lnc215*	0.61	3.11	0.84	2.25	0.03	0.00	1.00	1.00	0.00	long non-coding RNA 215
*Nabp1*	1.08	3.03	7.05	0.46	0.89	0.04	1.00	1.00	0.01	nucleic acid binding protein 1, transcript variant X1
*Cryl1*	0.55	2.78	1.99	0.77	0.35	0.05	1.00	1.00	0.07	crystallin, lambda 1, transcript variant X1
*Map3k7cl*	0.90	2.70	1.59	1.53	0.56	0.02	1.00	1.00	0.00	MAP3K7 C-terminal like, transcript variant X1
*Fah*	0.57	2.67	1.96	0.78	0.12	0.02	1.00	1.00	0.00	fumarylacetoacetate hydrolase
*Amigo2*	0.99	2.66	0.92	2.88	0.96	0.03	1.00	1.00	0.00	adhesion molecule with Ig like domain 2, transcript variant X3
*Utrn*	0.75	2.64	2.18	0.91	0.15	0.03	1.00	1.00	0.00	utrophin, transcript variant X3
*Zeb2*	0.47	2.61	0.46	2.69	0.32	0.04	1.00	1.00	0.08	zinc finger E-box binding homeobox 2, transcript variant X1
*Trmt61a*	0.52	2.59	1.02	1.32	0.13	0.02	1.00	1.00	0.01	tRNA methyltransferase 61A, transcript variant X1
*Ide*	0.61	2.47	1.68	0.89	0.08	0.03	1.00	1.00	0.01	insulin degrading enzyme, transcript variant X1
*Hsd17b12*	0.61	2.40	1.00	1.47	0.14	0.03	1.00	1.00	0.01	hydroxysteroid (17-beta) dehydrogenase 12, transcript variant X1
*Il34*	1.24	2.40	1.01	2.92	0.27	0.01	1.00	1.00	0.00	interleukin 34, transcript variant X1
*C1s*	0.81	2.39	1.44	1.35	0.21	0.03	1.00	1.00	0.00	complement component 1, s subcomponent
*RragB*	0.70	2.38	0.94	1.77	0.37	0.03	1.00	1.00	0.05	Ras-related GTP binding B, transcript variant X2
*Smim12*	0.61	2.38	0.91	1.60	0.01	0.02	1.00	1.00	0.00	small integral membrane protein 12, transcript variant X1
*Mvd*	0.82	2.31	0.75	2.53	0.49	0.04	1.00	1.00	0.02	mevalonate diphosphate decarboxylase
*Pld2*	0.88	2.27	0.72	2.78	0.50	0.03	1.00	1.00	0.01	phospholipase D2, transcript variant X1
*Aox1*	0.78	2.27	1.64	1.08	0.54	0.04	1.00	1.00	0.06	aldehyde oxidase 1
*Fos*	0.34	2.23	0.28	2.68	0.07	0.04	1.00	1.00	0.00	FBJ osteosarcoma oncogene
*Mal*	0.83	2.23	0.86	2.15	0.67	0.03	1.00	1.00	0.10	mal, T-cell differentiation protein
*Gemin8*	0.50	2.21	0.68	1.63	0.33	0.02	1.00	1.00	0.19	gem (nuclear organelle) associated protein 8
*Yif1a*	0.85	2.20	2.00	0.94	0.32	0.02	1.00	1.00	0.00	Yip1 interacting factor homolog A, membrane trafficking protein
*Ppdpf*	0.49	2.10	0.96	1.08	0.01	0.00	1.00	1.00	0.00	pancreatic progenitor cell differentiation and proliferation factor, transcript variant X2
*Lcp1*	0.69	2.10	1.09	1.32	0.66	0.05	1.00	1.00	0.54	lymphocyte cytosolic protein 1, transcript variant X2
*Slc35f6*	0.85	2.08	1.06	1.67	0.63	0.01	1.00	1.00	0.07	solute carrier family 35, member F6
*Gpd1*	0.86	2.05	1.11	1.59	0.22	0.01	1.00	1.00	0.00	glycerol-3-phosphate dehydrogenase 1, transcript variant X1
*Dchs1*	1.35	2.04	1.51	1.83	0.23	0.02	1.00	1.00	0.00	dachsous cadherin-related 1, transcript variant X1
*Rrs1*	0.92	2.02	1.90	0.98	0.86	0.04	1.00	1.00	0.14	ribosome biogenesis regulator homolog
*Chchd6*	0.79	2.00	0.84	1.89	0.12	0.04	1.00	1.00	0.01	coiled-coil-helix-coiled-coil-helix domain containing 6

**Table 2:** List of downregulated genes in cochlear nuclei of rats treated with noise and fluoxetine compared to noise exposure alone.

Gene symbol	Fold change				p-value					Gene description
	Noise+vehicle/vehicle	Noise+fluoxetine/Noise+vehicle	Fluoxetine/vehicle	Noise+fluoxetine/fluoxetine	Noise+vehicle/vehicle	Noise+fluoxetine/Noise+vehicle	Fluoxetine/vehicle	Noise+fluoxetine/fluoxetine	ANOVA	
*Anks1b*	1.02	0.50	0.90	0.56	0.80	0.02	1.00	1.00	0.00	ankyrin repeat and sterile alpha motif domain containing 1B
*Bicd1*	1.27	0.48	0.83	0.74	0.09	0.05	1.00	1.00	0.03	BICD cargo adaptor 1, transcript variant X1
*Kdm1b*	1.33	0.48	0.36	1.77	0.06	0.05	1.00	1.00	0.01	lysine demethylase 1B
*Copb1*	0.86	0.48	0.89	0.46	0.11	0.02	1.00	1.00	0.00	coatomer protein complex, subunit beta 1
*Zcchc11*	0.84	0.48	1.03	0.39	0.30	0.04	1.00	1.00	0.02	zinc finger CCHC-type containing 11
*Aurkc*	1.32	0.47	0.84	0.74	0.27	0.01	1.00	1.00	0.03	aurora kinase C
*Alg6*	1.75	0.47	1.38	0.60	0.05	0.01	1.00	1.00	0.02	ALG6, alpha-1,3-glucosyltransferase
*Npat*	1.04	0.47	0.76	0.65	0.25	0.01	1.00	1.00	0.00	nuclear protein, co-activator of histone transcription
*Tsfm*	0.88	0.47	0.98	0.42	0.62	0.03	1.00	1.00	0.03	Ts translation elongation factor, mitochondrial
*Sept6*	1.13	0.47	0.55	0.96	0.33	0.03	1.00	1.00	0.00	septin 6, transcript variant X3
*Pnpt1*	0.88	0.46	0.63	0.64	0.68	0.02	1.00	1.00	0.05	polyribonucleotide nucleotidyltransferase 1
*Nupl2*	1.35	0.45	1.09	0.56	0.06	0.04	1.00	1.00	0.05	nucleoporin like 2
*Xkr8*	2.24	0.45	1.62	0.62	0.07	0.03	1.00	1.00	0.06	XK related 8
*Kcnma1*	1.12	0.45	0.43	1.17	0.22	0.01	1.00	1.00	0.00	potassium calcium-activated channel subfamily M alpha 1
*Zfp280d*	0.71	0.45	0.73	0.43	0.01	0.00	1.00	1.00	0.00	zinc finger protein 280D, transcript variant X1
*Zcchc8*	0.94	0.44	0.72	0.57	0.86	0.02	1.00	1.00	0.05	zinc finger CCHC-type containing 8, transcript variant X2
*Brinp1*	0.94	0.44	0.86	0.48	0.86	0.02	1.00	1.00	0.07	BMP/retinoic acid inducible neural specific 1, transcript variant X1
*Dlgap3*	1.50	0.43	1.14	0.57	0.09	0.03	1.00	1.00	0.00	DLG associated protein 3, transcript variant 1
*Rbm12*	1.72	0.43	1.39	0.53	0.07	0.00	1.00	1.00	0.02	RNA binding motif protein 12, transcript variant X2
*Morc3*	1.04	0.43	1.08	0.41	0.47	0.01	1.00	1.00	0.00	MORC family CW-type zinc finger 3, transcript variant X1
*Nle1*	1.75	0.43	0.89	0.83	0.02	0.01	1.00	1.00	0.00	notchless homolog 1, transcript variant X2
*Bcl9l*	1.79	0.42	1.00	0.75	0.22	0.02	1.00	1.00	0.23	B-cell CLL/lymphoma 9-like
*Pex26*	0.98	0.42	0.70	0.58	0.90	0.04	1.00	1.00	0.03	peroxisomal biogenesis factor 26, transcript variant X1
*Ppp4c*	0.70	0.40	0.66	0.43	0.08	0.00	1.00	1.00	0.00	protein phosphatase 4, catalytic subunit, transcript variant X1
*Zfp426*	1.17	0.40	0.70	0.67	0.57	0.02	1.00	1.00	0.03	zinc finger protein 426, transcript variant X9
*Aldh4a1*	1.38	0.40	1.04	0.53	0.01	0.01	1.00	1.00	0.00	aldehyde dehydrogenase 4 family, member A1
*Cap2*	1.09	0.40	0.79	0.54	0.70	0.00	1.00	1.00	0.01	CAP, adenylate cyclase-associated protein, 2 (yeast), transcript variant X2
*Kcng4*	0.88	0.39	0.50	0.69	0.29	0.03	1.00	1.00	0.01	potassium voltage-gated channel modifier subfamily G member 4
*Trappc8*	0.76	0.39	0.95	0.31	0.38	0.04	1.00	1.00	0.01	trafficking protein particle complex 8, transcript variant X1
*Zfp667*	1.17	0.39	1.13	0.40	0.43	0.01	1.00	1.00	0.00	zinc finger protein 667
*Clp1*	2.27	0.38	0.89	0.98	0.06	0.05	1.00	1.00	0.00	cleavage and polyadenylation factor I subunit 1, transcript variant X3
*Fam175a*	1.96	0.38	1.56	0.48	0.00	0.03	1.00	1.00	0.03	family with sequence similarity 175, member A
*Slc25a16*	1.02	0.38	1.10	0.35	0.94	0.01	1.00	1.00	0.02	solute carrier family 25 member 16
*Zfp207*	0.93	0.36	0.80	0.42	0.77	0.01	1.00	1.00	0.01	zinc finger protein 207, transcript variant X4
*Hook1*	1.02	0.35	1.15	0.31	0.95	0.01	1.00	1.00	0.01	hook microtubule-tethering protein 1
*Rn5-8s*	1.02	0.34	0.69	0.50	0.96	0.01	1.00	1.00	0.07	5.8S ribosomal RNA
*Nhlh2*	0.93	0.34	0.88	0.35	0.84	0.04	1.00	1.00	0.09	nescient helix loop helix 2, transcript variant X1
*Pcgf6*	0.65	0.33	0.47	0.45	0.27	0.03	1.00	1.00	0.00	polycomb group ring finger 6, transcript variant X2
*Rn5-8s*	0.99	0.32	0.70	0.45	0.98	0.02	1.00	1.00	0.07	5.8S ribosomal RNA
*Cnnm1*	2.43	0.31	0.92	0.81	0.02	0.01	1.00	1.00	0.00	cyclin and CBS domain divalent metal cation transport mediator 1, transcript variant X2
*Atxn2l*	0.90	0.30	0.82	0.33	0.69	0.02	1.00	1.00	0.00	ataxin 2-like, transcript variant X3
*Rn5-8s*	1.02	0.29	0.71	0.42	0.94	0.01	1.00	1.00	0.04	5.8S ribosomal RNA
*Rn5-8s*	1.01	0.28	0.73	0.39	0.99	0.01	1.00	1.00	0.04	5.8S ribosomal RNA
*Ccnl2*	1.14	0.28	0.73	0.43	0.63	0.05	1.00	1.00	0.01	cyclin L2, transcript variant X1
*Nr4a1*	0.76	0.28	0.69	0.31	0.04	0.01	1.00	1.00	0.00	nuclear receptor subfamily 4, group A, member 1, transcript variant X2
*Pik3r1*	1.98	0.28	1.75	0.31	0.08	0.01	1.00	1.00	0.02	phosphoinositide-3-kinase regulatory subunit 1, transcript variant X1
*Ap3m2*	1.37	0.26	1.44	0.25	0.07	0.00	1.00	1.00	0.00	adaptor-related protein complex 3, mu 2 subunit
*Ldb1*	1.00	0.26	0.83	0.31	1.00	0.04	1.00	1.00	0.03	LIM domain binding 1, transcript variant X4
*Casc3*	1.68	0.25	1.01	0.41	0.15	0.04	1.00	1.00	0.01	cancer susceptibility candidate 3
*Slc18a2*	0.76	0.25	0.29	0.66	0.21	0.03	1.00	1.00	0.03	solute carrier family 18 member A2, transcript variant X1
*Dgkg*	1.24	0.22	0.58	0.48	0.11	0.04	1.00	1.00	0.10	diacylglycerol kinase, gamma, transcript variant X3
*Pde5a*	1.48	0.09	1.00	0.14	0.07	0.00	1.00	1.00	0.00	phosphodiesterase 5A, transcript variant X1

**Fig 7:**
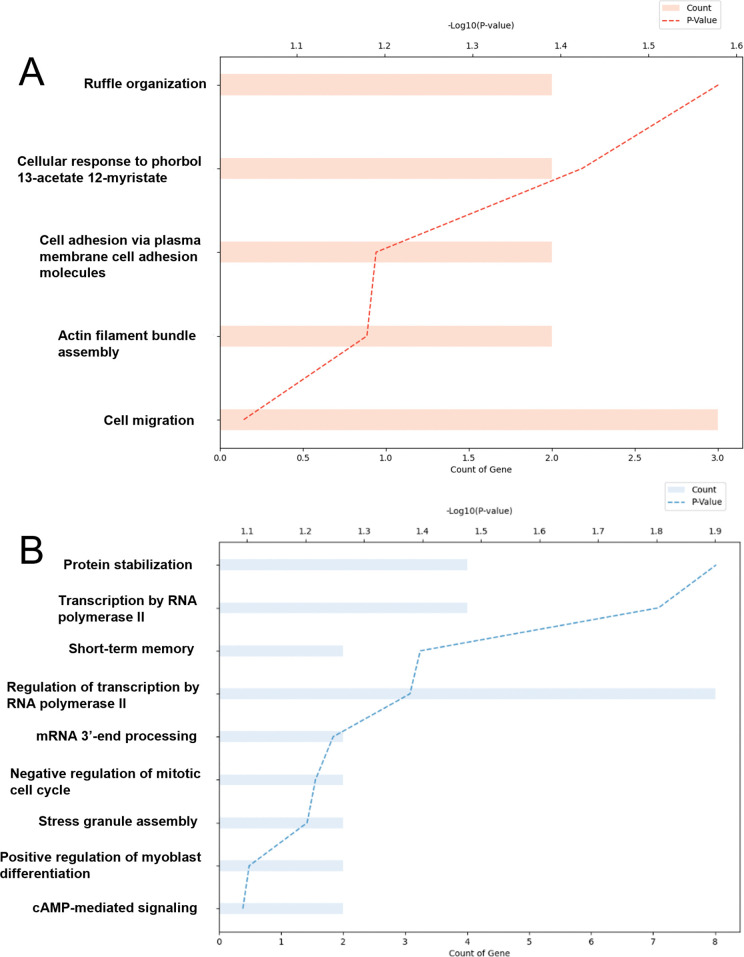
Gene Ontology Biological Process (GOBP) analysis revealed differential pathway regulation in noise+fluoxetine rats compared to noise+vehicle rats. (A) Pathways related to ruffle organization and cellular response to phorbol 13-acetate 12-myristate were predicted to be upregulated in the noise+fluoxetine group. (B) Pathways involved in protein stabilization, transcription by RNA polymerase II, short-term memory, and regulation of transcription by RNA polymerase II were predicted to be downregulated in the noise+fluoxetine group.

Several biological processes were found to be significantly enriched under different treatment groups, as revealed by Gene Ontology Biological Process (GOBP) analysis (Fig 7). Notably, processes such as ruffle organization and cellular response to phorbol 13-acetate 12-myristate were significantly enriched, with ruffle organization showing the highest log₁₀(p-value) and fold enrichment (Fig 7A). On the other hand, genes involved in protein stabilization, transcription by RNA polymerase II, short-term memory, and regulation of transcription by RNA polymerase II were significantly downregulated according to the GOBP analysis (Fig 7B).

## 4. Discussion

Fluoxetine administration in noise-induced hearing loss rats did not recover the cochlear damage and auditory threshold in the present study. However, fluoxetine reduced PNNs and was accompanied by transcriptional changes in the VCN. In the VCN, noise+fluoxetine rats presented decreased expression of ACAN and upregulated genes related to the ruffle organization and downregulated genes related with protein stabilization. In summary, fluoxetine administration promoted extracellular matrix remodeling and cytoskeletal reorganization in the VCN, but did not reverse noise‑induced cochlear damage or normalize ABR thresholds. Thus, the present data indicate a dissociation between central molecular plasticity and peripheral auditory function. Fluoxetine had minimal effect on cochlear injury caused by noise exposure, and its TrkB‑mediated actions appear insufficient for cochlear protection. These findings support a role for fluoxetine in driving central remodeling rather than peripheral auditory recovery.

Although fluoxetine produced a statistically significant improvement in ABR thresholds at low frequencies in noise‑exposed rats, the effect sizes were modest and restricted to 4 kHz when compared with noise‑only animals, and thresholds remained elevated relative to controls. Consistent with the unchanged hair‑cell morphology and extracellular baskets in the cochlea, these data indicate that fluoxetine does not provide robust peripheral auditory recovery but rather exerts limited, frequency‑specific effects on ABR thresholds. These results suggest that the effect of fluoxetine on hearing loss may differ from that of direct TrkB agonists. Although fluoxetine has been shown to bind to TrkB receptors [15], its TrkB-mediated activation may be insufficient to achieve robust auditory protection. In contrast, TrkB agonists have been reported to restore cochlear ribbon synapses in noise-induced synaptopathy [17]. In the present study, the expression of ACAN and BCAN in the extracellular baskets surrounding inner hair cells—structures analogous to PNNs in the central nervous system—was reduced following noise exposure and not restored by fluoxetine treatment. ACAN and BCAN are chondroitin sulfate proteoglycans that are key components of PNNs and may contribute to synaptic stabilization in both the cochlear and central auditory pathways [30,33]. In addition, a previous study reported that fluoxetine has been shown to enhance experience‑dependent plasticity in adult visual cortex by facilitating serotonin‑dependent modulation of orientation tuning in V1 neurons [34]. Thus, fluoxetine may act on the central auditory nervous system to rejuvenate neural plasticity.

In VCN, fluoxetine administration attenuated the expression of PNNs in this study. The results of the present study are in line with those of previous studies, which demonstrated decreased PNN expression facilitates neural plasticity. In PV interneurons of the visual cortex, fluoxetine administration induced critical period-like plasticity by facilitating TrkB phosphorylation and attenuating PNN, including ACAN [22]. BNDF activates TrkB by inducing its phosphorylation, thereby initiating downstream signaling cascades associated with synaptic plasticity. Protein tyrosine phosphatase σ (PTPσ), a known receptor for PNNs, interacts with TrkB to inhibit its activation. Upon TrkB phosphorylation, this interaction is disrupted, allowing for the activation of plasticity-related pathways [22]. Consistent with this, both pharmacological and optogenetic activation of TrkB have been shown to attenuate PNNs surrounding PV interneurons [12]. In the auditory system, fluoxetine was previously reported to reduce PNN expression in the primary auditory cortex, and sound enrichment enhanced auditory processing in noise-exposed rats [11]. However, the effects of fluoxetine on the peripheral auditory system, particularly the VCN, remain largely unexplored. In the present study, fluoxetine treatment in noise-exposed rats led to reduced expression of PNNs in the VCN. This reduction may facilitate enhanced neural plasticity in this region. Although BDNF mRNA levels were not significantly altered following fluoxetine treatment, transcriptomic changes indicative of altered plasticity-related pathways were observed in the VCN of fluoxetine-treated rats. In addition, changes in extracellular baskets may influence auditory cortical processing by modulating synaptic input, thereby altering the balance of excitation and inhibition that shapes cortical responses [35,36]. Such ECM remodeling may remove structural brakes on cortical plasticity and thereby reopen critical‑period–like plasticity, allowing experience‑dependent reorganization of tonotopic maps in the auditory cortex.

In the VCN, several fluoxetine‑responsive genes identified in our RNA‑seq data are closely linked to established pathways of neural plasticity and synaptic remodeling. For example, *Fos* is an immediate early gene that integrate activity‑dependent inputs to regulate cytoskeletal dynamics and structural synaptic changes [37], while *Adamts4* is an extracellular matrix–modifying protease that can cleave chondroitin sulfate proteoglycans such as aggrecan, thereby loosening perineuronal nets [38]. Together with the Gene Ontology enrichment for ruffle organization and actin‑based cytoskeletal remodeling, these transcriptional changes are consistent with a shift toward a more plastic, structurally labile state in the cochlear nuclei. In addition, genes related to the neuropeptide signaling pathway, including *Adcyap1, Nts, Npy*, and *Glra1*, were predicted to be increased in the VCN of noise+fluoxetine rats compared with those of noise rats. *Adcyap1* has been reported to regulate homeostatic activities in response to external stimuli, such as caloric restriction and inflammation [39,40]. *Nts* has been suggested to regulate a variety of aspects of reward processes in the brain and midbrain regions [41]. The auditory nervous system also has neurotensin-expressing neurons whose role in noise and fluoxetine treatment needs to be explored in further studies [42]. *Npy* is associated with short-term synaptic plasticity and modulates recurrent excitatory activities in the inferior colliculi, which are mostly expressed in glutamatergic neurons [43]. *Npy* has also been suggested as a protective signal in olivocochlear neurons [44]. Thus, the increase in *Npy* in noise+fluoxetine-treated rats in the present study may indicate that synaptic plasticity protects cochlear neurons. *Glra1* is an inhibitory receptor in cochlear nuclei whose expression is decreased after auditory deafferentiation [45]. Our findings of increased *Glra1* after fluoxetine treatment indicated enhanced inhibitory auditory neural signals in cochlear nuclei. On the other hands, genes related with cellular potassium ion homeostasis and response to activity were predicted to be decreased in noise+fluoxetine rats which were increased in noise rats. Because potassium ion transporting regulates action potential firing in neurons of cochlear nuclei, modulation of this pathway may contribute to the fluoxetine-induced neural plasticity in the current study [46]. For the genes related with response to activity, *Ccl5* was reported to be increased in cochleae with oxidative stress [47]. *Nos1* was previously reported to be expressed in multiple auditory centers including type I cochlear afferents and auditory brainstems [48,49]. In addition, production of NO by NOS regulated synaptic plasticity and suppress excitatory activity driven by N-Methyl-D-aspartic acid [50]. Thus, *Ccl5* and *Nos1* may promoted noise-induced neural damage, which were alleviated after fluoxetine treatment.

This study has several limitations. First, the conditions of early-life noise exposure and fluoxetine administration may not fully reflect the complexity of auditory development. Second, although the effects of fluoxetine were presumed to be mediated via TrkB activation, we did not directly measure TrkB phosphorylation or employ TrkB antagonists, limiting the ability to confirm a causal relationship. Third, RNA sequencing was performed with a limited sample size, which may affect the statistical robustness of differential gene expression analysis. Thus, the functional relevance of DEGs should be interpreted with caution and considered as exploratory findings that primarily inform central plasticity rather than peripheral recovery. Further studies are needed to elucidate the molecular pathways associated with the genes predicted in the present study.

In conclusion, this study demonstrates that noise-induced hearing loss leads to a decrease in ACAN and BCAN expression in the extracellular baskets of inner hair cells, with corresponding reductions in PNNs in the VCN. Fluoxetine administration induced central changes, including reduced ACAN‑positive PNNs in the VCN and transcriptional alterations related to cytoskeletal and extracellular matrix remodeling, but produced only limited and frequency‑restricted improvements in ABR thresholds and did not restore cochlear structure. Overall, fluoxetine appears to drive central remodeling and transcriptional plasticity without providing robust peripheral auditory protection, indicating that TrkB signaling engaged by fluoxetine is not sufficient for cochlear rescue and may differ mechanistically from direct TrkB agonists. These results temper expectations for fluoxetine as a stand‑alone otoprotective therapy and instead highlight its value for probing central plasticity mechanisms in noise‑induced hearing loss.
